# Prophylactic antibiotic treatment with TMP-SMX decreased the incidence of interstitial pneumonia in patients with B-cell lymphoma on chemotherapy

**DOI:** 10.1186/s12885-020-07254-w

**Published:** 2020-08-08

**Authors:** Cong Li, Fangxiao Lu, Tao Lei, Haifeng Yu, Xi Chen, Shuailing Peng, Shuiyun Han, Haiyan Yang

**Affiliations:** 1 Department of Medical Oncology, Institute of Cancer and Basic Medicine (ICBM), Chinese Academy of Sciences, Hangzhou, China; 2 Department of Medical Oncology, Cancer Hospital of the University of Chinese Academy of Sciences, Hangzhou, China; 3 Department of Medical Oncology, Zhejiang Cancer Hospital, Hangzhou, China; 4 Department of Medical Imaging, Zhejiang Cancer Hospital, Hangzhou, China

**Keywords:** B-cell lymphoma, Chemotherapy, Interstitial pneumonia, TMP-SMX, Rituximab

## Abstract

**Background:**

Several studies have reported the incidence of interstitial pneumonia (IP) among patients with non-Hodgkin lymphoma (NHL) that are undergoing combination chemotherapy plus rituximab; however, the effective prophylactic treatment for IP remains unclear. This study aims to explore the prophylactic effect of trimethoprim-sulfamethoxazole (TMP-SMX) on IP and identify IP-associated risk factors in NHL patients.

**Methods:**

Between March 2013 and April 2018, 498 patients (264 males, 53%) with B-cell NHL undergoing first-line RCHOP-like chemotherapy treatment with rituximab plus cyclophosphamide, doxorubicin, vincristine, and prednisone were enrolled in this study.

**Results:**

These patients had a median age of 56 years, and 311 of the 498 patients (62.4%) were administered once daily with the prophylactic treatment of TMP-SMX. IP occurred in 65 patients (13.1%), indicating a significant reduction in the IP incidence rate (21.4% vs. 8.0%; *p* < 0.001). Among patients treated with TMP-SMX, 2 (1.2%) exhibited rashes, 38 (12.2%) suffered from nausea and vomiting, 52 (16.7%) showed signs of neutropenia, and 18 (5.8%) suffered from kidney dysfunction. Both univariate and multivariate analysis showed that gender (male), history of diabetes, and absence of prophylactic TMP-SMX treatment were significant risk factors associated with IP. Disease progression was observed in 55/311 (17.7%) patients that underwent prophylactic TMP-SMX treatment and in 63/187 (33.7%) patients that did not (*p* < 0.001).

**Conclusions:**

This study revealed that the occurrence of IP was common in B-cell NHL patients undergoing combined chemotherapy plus rituximab treatment. IP could be reduced with prophylactic treatment of once-daily oral TMP-SMX.

## Background

The RCHOP regimen has been widely employed for the treatment of CD20+ non-Hodgkin lymphoma (NHL) [1–3]. Previous studies have reported that while receiving immunochemotherapy, approximately 1.3 to 14% of the patients develop interstitial pneumonia (IP) [4–8]. Individuals diagnosed with IP usually stop further chemotherapy, thereby reducing its efficiency and shortening patient survival [9]. Therefore, active treatment and prevention of IP are essential for patients undergoing immunochemotherapeutic treatment. However, the most effective prophylactic drugs and methods to prevent IP remains controversial. Several studies have concluded that the occurrence of IP is partially associated with a rising risk for pneumocystis carinii pneumonia (PCP) infection [4–6, 10, 11]. Trimethoprim-sulfamethoxazole (TMP-SMX) is a prophylactic drug that is specifically used for the treatment of PCP infections [5, 6, 11]. In this study, we present a retrospective analysis of the impact of TMP-SMX prophylaxis on IP incidences among B-cell NHL patients undergoing combination chemotherapy plus rituximab.

## Methods

### Study participants and data collection

Study inclusion criteria were as follows: patients who were diagnosed with CD20+ B-cell NHL based on WHO criteria [12], and those who had received RCHOP-like chemotherapy for at least two cycles. Exclusion criteria were as follows: patients with T cell lymphomas and patients without full treatment data were excluded. TMP-SMX was administered once daily, from the time of treatment initiation until completion of chemotherapy. Each tablet contained 0.08 g TMP and 0.4 g SMX. Radiographic documents and clinical data regarding patient characteristics, histological diagnoses, chemotherapy regimens, and survival outcomes were retrospectively collected.

### IP diagnosis and treatment

The observation period for IP started from the first day of immunochemotherapy and lasted 8 months after completion of immunochemotherapy. Routine imaging evaluations were performed every two cycles during chemotherapy and every 3 months after chemotherapy. Computed tomography (CT) scans of the thoracic cavity were collected when patients exhibited symptoms of pulmonary infection. IP was diagnosed via a multidisciplinary approach based on clinical symptoms, laboratory tests, radiologic imaging, and pathologic findings. IP typically presented in the form of diffused pulmonary interstitial infiltrates with reticular or ground-glass opacity, alveolitis, and the presence of diffused infiltrates on the CT scans [11, 13]. When IP was suspected, laboratory tests were performed to measure blood cell counts, inflammatory indicators, and bacterial culture. Once clinically diagnosed with IP, patients began undergoing empirical treatment with a combination of antibiotics, antifungal agents, and glucocorticoids. Ganciclovir was given if a viral infection was suspected. When PCP was suspected, a therapeutic dose of TMP-SMX was administered. CT scans were conducted weekly until complete absorbance of interstitial infiltrate was achieved. After patients recovered from IP, retreatment with chemotherapy and/or rituximab was allowed.

### Statistical analysis

Overall survival (OS) was calculated from the administration of the first chemotherapy to death or last contact. Progression-free survival (PFS) was calculated from the first administration of chemotherapy to disease progression, relapse, or death, whichever occurred first. The National Cancer Institute Common Terminology Criteria for Adverse Events (NCI-CTCAE) v4.0 criteria were used for the gradation of all adverse events (AEs). χ2 tests were used to compare the categorical variables between patient groups. A binary logistic regression was used to conduct univariate analyses with appropriate hazard ratios (HRs) and 95% confidence intervals (CIs). Each variable with a *p* < 0.05 in the initial univariate analysis was incorporated into the multivariable model. SPSS 23.0 was used for all statistical testing.

## Results

### Patient characteristics

Between March 2013 and April 2018, a total of 498 patient data was analyzed in this study, of whom the majority (264/498; 53%) were male. Table 1 compiles the clinical characteristics of these patients. These patients had a median age of 56 years (range: 18–82). Of the 498 patients, 414 (83.1%) were diagnosed with diffuse large B-cell lymphoma (DLBCL), 9 (1.8%) with mantle cell lymphoma (MCL), 36 (7.2%) with follicular lymphoma (FL), 7 (1.7%) with chronic lymphocytic leukemia/small B-cell lymphoma (CLL/SLL), 25 (5%) with marginal zone lymphoma (MZL), and 7 (1.7%) with highly aggressive B-cell lymphoma. The most common chemotherapy regimens included RCHOP (*n* = 428, 85.9%), rituximab plus cyclophosphamide, doxorubicin, vincristine, prednisone, and etoposide (REPOCH) (*n* = 57, 11.4%), and rituximab plus cyclophosphamide, vincristine, and prednisone (RCOP) (*n* = 13, 2.6%). Three hundred and eleven patients (62.4%) were administered TMP-SMX, while the remaining 187 patients (37.6%) did not undergo any prophylactic treatment. Except for gender, other baseline characteristics did not differ significantly between groups.

**Table 1:** Baseline characteristics of all the patients (*n* = 498)

Factor		All patients (%)	prophylaxis of TMP/SMX		*p*
			Yes	No	
Age	> 60 years	182 (36.5%)	112 (61.5%)	70 (38.5%)	0.774
	≤ 60 years	316 (63.5%)	199 (63%)	117 (37%)	
Gender	Female	234 (47%)	158 (67.5%)	76 (32.5%)	0.033
	Male	264 (53%)	153 (58%)	111 (42%)	
ECOG	PS > 1	57 (11.4%)	42 (73.7%)	15 (26.3%)	0.08
	PS ≤1	441 (88.6%)	269 (61%)	172 (39%)	
Elevated LDH	YES	231 (46.4%)	145 (62.8%)	86 (37.2%)	0.926
	NO	267 (53.6%)	166 (62.2%)	101 (37.8%)	
Smoking history	YES	131 (26.3%)	77 (58.8%)	54 (41.2%)	0.344
	NO	367 (73.7%)	234 (63.8%)	133 (36.2%)	
Ann Arbor stage	I	88 (17.7%)	45 (51.1%)	43 (48.9%)	0.071
	II	162 (32.5%)	103 (63.6%)	59 (36.4%)	
	III	97 (19.5%)	60 (61.9%)	37 (38.1%)	
	IV	151 (30.3%)	103 (68.2%)	48 (31.8%)	
BM involvement	YES	27 (5.4%)	19 (70.4%)	8 (29.6%)	0.422
	NO	471 (94.6%)	292 (62%)	179 (38%)	
IPI risk (score)	Low (0–1)	236 (47.4%)	140 (59.3%)	96 (40.7%)	0.378
	Low–intermediate-2	126 (25.3%)	81 (64.3%)	45 (35.7%)	
	High–intermediate-3	80 (16.1%)	50 (62.5%)	30 (37.5%)	
	High (4–5)	56 (11.2%)	40 (71.4%)	16 (28.6%)	

### Diagnosis and treatment of IP

IP occurred in 65 patients (13.1%), of whom 25 (38.5%) were in the TMP-SMX prophylaxis group. IP patients had a median age of 60 years (range: 18 to 78) and exhibited the following pathological diagnoses: 57 cases of DLBCL, 1 case of FL, 1 case of MCL, 1 case of CLL/SLL, 3 cases of MZL, and 2 cases of highly aggressive B-cell lymphoma. IP incidence was significantly higher among the patients who had not undergone prophylactic TMP-SMX treatment relative to those who did (21.4% vs. 8.0%, *p* < 0.001). The median time between the first day of immunochemotherapy and the date of IP diagnosis was 74 days (range: 7–158 days). Before IP diagnosis, patients had been treated with a median of 3 cycles (range: 1 to 8 cycles). The median cumulative dose of rituximab on IP diagnosis was 2100 mg (range: 600 to 4800 mg). Table 2 shows a review of these 65 cases.

**Table 2:** Review of 65 cases with interstitial pneumonia

Factor		Number
Age	> 60 years	30 (46.2%)
	≤60 years	35 (35.8%)
Sex	Female	23 (35.4%)
	Male	42 (64.6%)
ECOG	> 1	3 (4.6%)
	≤1	62 (95.4%)
Elevated LDH	YES	29 (44.6%)
	NO	36 (55.4%)
Smoking history	YES	23 (35.4%)
	NO	42 (64.6%)
Ann Arbor stage	I	12 (18.5%)
	II	24 (36.9%)
	III	15 (23.1%)
	IV	14 (21.5%)
IPI risk (score)	Low (0–1)	34 (52.3%)
	Low–high (2–3)	26 (40.0%)
	High (4–5)	5 (7.7%)
Diabetes history	YES	12 (18.5%)
	NO	53 (81.5%)

Fifteen (23.1%) patients exhibited no IP-associated symptoms, although a routine radiological examination detected IP. Common clinical presentation of IP included cough (*n* = 24), fever (*n* = 31), chest distress (*n* = 20), expectoration (*n* = 17), and shortness of breath (*n* = 11). Elevated levels of C-reactive protein (CRP) (> 10 mg/L) and procalcitonin (PCT) (> 0.5 ng/mL) were evident in 54 and 5 patients, respectively. Eighteen patients showed a positive G test for 3-b-D glucan fungal antigens (> 60 pg/mL). Elevated levels of Gram-negative lipopolysaccharides (> 10 pg/mL) were detected in 36 cases. Type I respiratory failure was confirmed in five patients based on arterial blood gas measurements. Sputum cultures were performed in patients following expectoration, and positive pathogenic bacteria included *Candida albicans* (*n* = 3), *Staphylococcus aureus* (*n* = 1), Klebsiella aeruginosa (*n* = 2), Klebsiella pneumonia (*n* = 2), hemolytic Streptococcus (*n* = 1), and Staphylococcus haemolyticus (*n* = 1). Negative blood cultures were obtained in eight patients with temperatures higher than 38.5 °C. Seven patients received bronchoalveolar lavage (BAL), but Pneumocystis carinii was detected in none of them. Other common suspected pathogens included haemophilus, stenotrophomonas, tropheryma, exophiala, and human gammaherpesvirus-4.

IP diagnosis resulted in immediate withholding of further chemotherapy and rituximab treatments. The average time for IP remission was 12 days (range: 7 to 58 days). There were no fatalities due to this infection. After remission from IP, 21 patients completed combined chemotherapy plus rituximab, 35 patients received chemotherapy without rituximab, and 9 patients did not receive either chemotherapy or rituximab. There were no cases of IP recurrence following continued immunochemotherapy treatment.

### Complications of prophylaxis

Of the 311 patients receiving prophylactic TMP-SMX, 2 (1.2%) had rashes, 38 (12.2%) suffered from nausea and vomiting, 52 (16.7%) exhibited neutropenia, and 18 (5.8%) suffered from kidney dysfunction. However, none of the patients discontinued TMP-SMX prophylactic treatment as a result of these adverse reactions. As these patients were undergoing concomitant chemotherapy, it was difficult to determine whether these adverse reactions were specifically associated with TMP-SMX prophylaxis.

### IP risk factors

Next, we tried to identify clinical risk parameters that were associated with IP (Table 3). Univariate analysis revealed that being a male, with a history of diabetes, and absence of TMP-SMX prophylactic therapy was associated with elevated risk for IP. In a subsequent multivariate analysis, all these three variables were found to predict a higher risk for IP independently.

**Table 3:** Binary logistic regression analysis of risk factors for interstitial pneumonia

Factor	Univariate analysis				Multivariate analysis			
	Odds ratio	*P* value	95%CI		Odds ratio	*P* value	95%CI	
			Lower	Upper			Lower	Upper
Male vs female	1.736	0.046	1.009	2.985	1.779	0.048	1.006	3.145
Age > 60 vs < =60	1.585	0.086	0.936	2.682				
IPI score > 2 vs < =2	1.587	0.159	0.835	3.018				
ECOG PS > 1 vs < =1	2.945	0.076	0.893	9.71				
Diabetes,yes or no	3.042	0.003	1.468	6.3	3.625	0.001	1.675	7.845
Smoking history, yes or no	1.648	0.077	0.948	2.865				
Baseline lung disease, yes or no	3.405	0.162	0.611	18.973				
Prophylactic TMP/SMX, yes or no	0.321	< 0.001	0.188	0.55	0.33	< 0.001	0.19	0.57
Elevated LDH, yes vs no	0.921	0.759	0.545	1.556				

### Impact of IP on survival

After a median 26-month follow-up period, median PFS and OS were 23.1 and 26.7 months, respectively. Among the 311 patients receiving prophylactic TMP-SMX treatment, 55 exhibited disease progression, while a significantly larger proportion of the patients who did not undergo prophylactic treatment (63/187) suffered from progressive disease (17.7% vs. 33.7%, *p* < 0.001).

## Discussion

Although immunochemotherapy-associated IP has been extensively studied; however, the exact incidence of IP among lymphoma patients remains unclear due to high data variability in previous studies. One retrospective analysis of 2212 Chinese lymphoma patients revealed an overall IP incidence rate of 3.75%, with 3.9% (7/287) and 2.4% (76/925) in patients with Hodgkin and NHL, respectively [9]. Giselle et al. and Wang et al. reported that IP incidence among NHL patients undergoing CHOP-based chemotherapy was 1.3 and 14.8%, respectively [7, 14]. Other groups have reported values within this range, including 4.4% (5/114 patients) by Toshiro Kurokawa et al. [15], 6.2% (8/129 patients) by Katsuya Hiroo el al [13]., 7% (5/71 patients) by Lim KH et al. [16], and 4.9% (26/529 patients) by Huang et al. [17]. These studies also suggested that the addition of the rituximab to therapeutic regimen might be responsible for the increase of IP incidence. In the present study, we observed a higher IP incidence rate of 21.4% in patients who did not receive preventive treatment, compared with previous reports. There could be several reasons for this difference. First, all patients received rituximab, which possessed broad immunomodulatory activity, thus, elevating the risk of opportunistic infection [18]. Second, these patients were actively monitored by CT, which may detect a higher number of asymptomatic patients. Other possible causes included differences in the baseline characteristics of the patient population, the chemotherapy regimens administered, the intensity of chemotherapeutic dosage, diagnostic techniques, or an extended observation period that was used in this study. Thus, our results suggest that IP is a common occurrence in NHL patients who are undergoing RCHOP therapy.

For patients undergoing immunochemotherapeutic treatment, opportunistic infections remain the leading cause of IP [19]. While all pathogens, including viruses, bacteria, and fungi, could potentially cause IP, PCP is one of the most prominent and deadly pathogens. TMP-SMX is a sulfa antibiotic that offers broad antibacterial efficacy [20]. TMP-SMX is the first-line agent used for PCP prophylaxis in HIV-infected individuals [21, 22]. Even among immunocompromised individuals who are HIV-negative, the preventive use of TMP-SMX during chemotherapy may decrease the incidence of PCP [15, 23]. Toshiro et al. found that the prophylactic administration of TMP-SMX to NHL patients, who are undergoing RCHOP-based treatment resulted in null cases of PCP infections [15]. With adequate drug adherence and tolerance, TMP-SMX prophylaxis has been shown to protect against 89% of PCP cases [24, 25]. Moreover, TMP-SMX is widely available and an inexpensive drug. Therefore, TMP-SMX was selected as the prophylactic agent in this study.

In 1977, Hughes et al. first demonstrated the successful use of TMP-SMX to treat pediatric cancer patients, where untreated patients had a 21% PCP incidence rate, and treated patients had a 0% PCP incidence rate when TMP-SMX was administered either daily or 3 days per week [26, 27]. More recently, many studies have confirmed the efficiency of TMP-SMX prophylaxis as a means of decreasing the PCP incidence rate [15, 28–31]. A meta-analysis of twelve randomized trials found that TMP-SMX administration was linked to a 91% drop in PCP incidence, with a significant reduction in PCP-related mortality [23].

However, the optimal administration schedule for prophylactic TMP-SMX treatment is not well-defined. In previous studies, TMP-SMX was administered either once daily [15], twice daily two times per week [30], two consecutive days per week [28, 32, 33], twice weekly [31], or 3 days per week [27]. A meta-analysis concluded that lower doses of TMP-SMX were an effective means of improving tolerance without compromising the prophylactic efficacy [23, 34]. Here, patients were administered one tablet of TMP-SMX per day, and this approach was convenient and easy to implement.

We found that a history of diabetes, being male, and not undergoing prophylactic TMP-SMX treatment were independent risk factors associated with IP. In diabetic patients, hyperglycemia affects the intracellular bactericidal efficacy of immune cells. Additionally, the thickening of the alveolar epithelium, degeneration of vascular hyaline, and pulmonary microangiopathy can affect lung function. Therefore, patients with diabetes had a 30% higher pneumonia-related mortality compared with non-diabetic patients [35]. Males usually receive higher doses of rituximab, have a longer smoking history, and are more likely to have poorer basic lung function than females. However, this study did not find other IP-associated risk factors that were identified in previous studies, such as the use of rituximab [13–15, 17, 36], pre-treatment absolute lymphocyte counts < 1 × 10^9^/L [17], B symptoms, a history of drug allergy [9, 14], and increased intensity of corticosteroid exposure [5, 37, 38].

This study had several limitations, which need to be considered while interpreting these results. There are several factors, other than infectious pathogens that can cause IP, such as environmental or chemical damage, or immune-mediated inflammation [19]. Our study observed that the prophylactic use of TMP-SMX decreased the IP incidence rate; hence, we speculated that TMP-SMX mainly decreased infection caused by PCP. However, currently, there is insufficient evidence regarding pathogens that cause IP. For example, BAL was not widely used in this study. Of the seven patients who received BAL, none of them suffered from PCP infections. Also, most patients were unwilling to receive biopsy of lung lesions. Additionally, the retrospective nature of this study increased the risk of unintentional bias, potentially explaining the observed discrepancies regarding the rate of side effects associated with TMP-SMX. Therefore, further prospective studies are needed to explore other prophylactic drugs and optimal administration.

## Conclusions

In conclusion, this study revealed that IP frequently occurred in B-cell NHL patients undergoing chemotherapy plus rituximab treatment. Prophylactic use of once-daily oral TMP-SMX could significantly reduce the IP incidence rate.

## Data Availability

All data generated during this study are included in this published article. The datasets used during the current study are available from the corresponding author on reasonable request.

## Abbreviations

TMP: Trimethoprim
SMX: Sulfamethoxazole
IP: Interstitial pneumonia
NHL: Non-Hodgkin lymphoma
RCHOP: Rituximab plus cyclophosphamide, doxorubicin, vincristine, and prednisone
PCP: Pneumocystis carinii pneumonia
G-CSF: Granulocyte colony-stimulating factor
CT: Computed tomography
CRP: C-reactive protein
PCT: Procalcitonin
GM: Galactomannan
OS: Overall survival
PFS: Progression-free survival
HRs: Hazard ratios
Cis: Confidence intervals
DLBCL: Diffuse large B cell lymphoma
MCL: Mantle cell lymphoma
FL: Follicular lymphoma
CLL/SLL: Chronic lymphocytic leukemia/small B cell lymphoma
MZL: Marginal zone lymphoma
REPOCH: Rituximab plus cyclophosphamide, doxorubicin, vincristine, prednisone, and etoposide
RCOP: Rituximab plus cyclophosphamide, vincristine, and prednisone
ECOG: Eastern cooperative oncology group
LDH: Lactate dehydrogenase
IPI: International prognostic index
BAL: Bronchoalveolar lavage
